# Assessing Barriers to Implementation of Machine Learning and Artificial Intelligence–Based Tools in Critical Care: Web-Based Survey Study

**DOI:** 10.2196/41056

**Published:** 2023-01-27

**Authors:** Eric Mlodzinski, Gabriel Wardi, Clare Viglione, Shamim Nemati, Laura Crotty Alexander, Atul Malhotra

**Affiliations:** 1 Division of Pulmonary, Critical Care, Sleep and Physiology University of California San Diego, CA United States; 2 Department of Emergency Medicine University of California San Diego, CA United States; 3 Dissemination and Implementation Science Center, Altman Clinical & Translational Research Institute University of California San Diego, CA United States; 4 Department of Biomedical Informatics University of California San Diego, CA United States; 5 Section of Pulmonary and Critical Care Veterans Affairs San Diego Healthcare System San Diego, CA United States

**Keywords:** surveys and questionnaires, machine learning, artificial intelligence, critical care, respiratory insufficiency, survey, Qualtrics, questionnaire, perception, trust, perspective, attitude, intubation, predict, barrier, adoption, implementation

## Abstract

**Background:**

Although there is considerable interest in machine learning (ML) and artificial intelligence (AI) in critical care, the implementation of effective algorithms into practice has been limited.

**Objective:**

We sought to understand physician perspectives of a novel intubation prediction tool. Further, we sought to understand health care provider and nonprovider perspectives on the use of ML in health care. We aim to use the data gathered to elucidate implementation barriers and determinants of this intubation prediction tool, as well as ML/AI-based algorithms in critical care and health care in general.

**Methods:**

We developed 2 anonymous surveys in Qualtrics, 1 single-center survey distributed to 99 critical care physicians via email, and 1 social media survey distributed via Facebook and Twitter with branching logic to tailor questions for providers and nonproviders. The surveys included a mixture of categorical, Likert scale, and free-text items. Likert scale means with SD were reported from 1 to 5. We used student t tests to examine the differences between groups. In addition, Likert scale responses were converted into 3 categories, and percentage values were reported in order to demonstrate the distribution of responses. Qualitative free-text responses were reviewed by a member of the study team to determine validity, and content analysis was performed to determine common themes in responses.

**Results:**

Out of 99 critical care physicians, 47 (48%) completed the single-center survey. Perceived knowledge of ML was low with a mean Likert score of 2.4 out of 5 (SD 0.96), with 7.5% of respondents rating their knowledge as a 4 or 5. The willingness to use the ML-based algorithm was 3.32 out of 5 (SD 0.95), with 75% of respondents answering 3 out of 5. The social media survey had 770 total responses with 605 (79%) providers and 165 (21%) nonproviders. We found no difference in providers’ perceived knowledge based on level of experience in either survey. We found that nonproviders had significantly less perceived knowledge of ML (mean 3.04 out of 5, SD 1.53 vs mean 3.43, SD 0.941; *P*<.001) and comfort with ML (mean 3.28 out of 5, SD 1.02 vs mean 3.53, SD 0.935; *P*=.004) than providers. Free-text responses revealed multiple shared concerns, including accuracy/reliability, data bias, patient safety, and privacy/security risks.

**Conclusions:**

These data suggest that providers and nonproviders have positive perceptions of ML-based tools, and that a tool to predict the need for intubation would be of interest to critical care providers. There were many shared concerns about ML/AI in health care elucidated by the surveys. These results provide a baseline evaluation of implementation barriers and determinants of ML/AI-based tools that will be important in their optimal implementation and adoption in the critical care setting and health care in general.

## Introduction

Machine learning (ML) is increasingly used for the development of predictive models in health care, although implementation into clinical care has been limited [1-5]. We have recently reported a deep learning algorithm to predict the need for intubation in patients at risk of respiratory failure in the intensive care unit (ICU) [6]. This algorithm was validated on multiple data sets and was shown to outperform expert clinicians as well as an established predictive model [7]. However, this algorithm is not yet widely implemented.

ML algorithms have been published across nearly all fields of medicine, with models developed for the interpretation of clinical imaging and pathology slides, to assist in the diagnosis of skin lesions, and to predict clinical decompensation and mortality risks in specific populations [8]. In the pulmonary and critical care space, there have been prediction models developed to identify and risk stratify pulmonary nodules on computed tomography scans, and sepsis prediction algorithms to detect clinical decompensation prior to patients meeting clinical sepsis criteria [9-12]. Mechanical ventilation is another area that has seen a growing number of algorithms, with particular focus on predicting successful weaning, ventilator-associated complications, ventilator asynchrony, and timing of need for intubation [3]. However, despite the novelty and potential utility of these models, most have not been used in patient care [1,2]. Thus, further efforts to elucidate barriers and facilitators to implementation are clearly warranted.

There have been a small number of studies evaluating perceptions of ML-based tools among providers or nonproviders using surveys or semistructured interviews. One study of 12 providers in the United Kingdom assessing perception of artificial intelligence (AI) in the National Health Services found concerns over a lack of infrastructure, funding, and a common language [4]. Another study of general practitioners in the United Kingdom suggested that these clinicians felt there was only limited potential for these technologies in their practice [13]. Richardson et al [14] assessed patient perceptions of AI in health care via semistructured interviews and found an overall positive perception of advances in health care technology but also concerns over safety, costs, and patient autonomy. Another study evaluating patient perceptions of ML/AI being used in a skin cancer screening tool found a favorable reception of this technology but only if being used to assist and not replace physician judgment [15]. A similar analysis of patient perception of ML/AI in skeletal radiography revealed a strong preference toward physician interpretation over an AI tool [16]. To our knowledge, there are no studies assessing potential implementation determinants of such tools in the critical care setting.

To identify barriers and facilitators to implementation of a novel tool that predicts the need for mechanical ventilation, as well as to better understand perceptions of ML-based tools across health care, we emailed surveys to providers and shared surveys via social media for both nonproviders and providers. We gathered qualitative information from the surveys to identify potential barriers, which may need to be addressed prior to optimal implementation of these approaches. We further sought to determine whether providers’ level of confidence with and perceived knowledge of ML would be a function of their level of experience. Overall, based on our experience and prior studies, we hypothesized that senior physicians may be less comfortable with ML algorithms compared to their more junior counterparts [16]. Second, we hypothesized that nonproviders would be more skeptical of machine learning tools than the providers who may be using them.

## Methods

### Survey Development

Our survey items were created and reviewed by a team of 4 critical care providers, 1 machine learning expert, and 1 implementation science expert to ensure completeness, functionality, and appropriate format based on published recommendations for surveys [17-21]. Survey structure and questions were not altered after survey dissemination. Respondents were provided with informed consent (Multimedia Appendices 1 and 2) and had the option to remain completely anonymous.

### Ethical Considerations

The University of California, San Diego (UCSD) institutional review board reviewed the study and waived the need for approval (UCSD IRB Project #210349XX, “Survey of ICU Clinicians Regarding the Implementation of a Novel EMR-Based Algorithm to Predict Need for Mechanical Ventilation in ICU Patients,” with an amendment for expanded survey with social media recruitment, initial waiver of approval date March 30, 2021, amendment waiver of approval date August 12, 2021).

### Single-Center Critical Care Physician Survey

Our single-center physician survey (Multimedia Appendix 1) was an open, voluntary, anonymous questionnaire that consisted of 8 items and was distributed to 99 critical care physician trainees and faculty at our institution via email. The survey consisted of 3 pages of content, with 6 multiple-choice and 2 free-response questions. Likert scales of 1-5 were used for opinion-based questions, with 1 representing the most negative and 5 the most positive outcome, and 3 representing a “moderate” response. The results are presented as means with SD. Likert scales were also converted to 3 groups with 1 and 2 representing “low,” 3 representing “moderate,” and 4 and 5 representing “high,” and the percentage of each category was reported. Respondents could go back and change answers prior to submitting the survey if desired. Data were collected over a 2-week period in May 2021.

### Social Media Survey

Our social media survey (Multimedia Appendix 2) was an open, voluntary, anonymous survey distributed via Twitter and Facebook posts (Multimedia Appendix 3) by our research team. The survey contained 3 pages of content and consisted of an initial question distinguishing medical providers from nonproviders, which then branched into an 11-question survey for providers and 10-question survey for nonproviders. Professions that were under the category of providers included: physicians (practicing or in-training), advanced practice providers, nurses, and medical students. Each survey included a mixture of multiple-choice and free-response questions. Likert scales of 1-5 were used for opinion-based questions, with 1 representing the most negative and 5 the most positive outcome, with 3 representing either a “neutral” or “moderate” response depending on the question. Outcomes are presented as means (SD). Likert scales were also converted to three groups with 1 and 2 representing “low” or “negative,” 3 representing “moderate” or “neutral,” and 4 and 5 representing “high” or “positive,” and the percentage of each category was reported. One adaptive question was used. Respondents could go back and change answers prior to submitting the survey if desired. Providers were offered the chance to complete the survey as a nonprovider as well, although this was not tracked. Data were collected over a 1-month period from September to October 2021. An incentive of an Amazon gift card was offered in the social media survey for one of the respondents chosen randomly.

### Survey Analyses

We used the CHERRIES (Checklist for Reporting Results of Internet E-Surveys) to guide our survey reporting and analysis (Multimedia Appendix 4) [22]. No view rate or participation rate was known for either survey. Data were collected and stored securely in Qualtrics for both surveys. The estimated time of each survey was approximately 5 minutes.

We performed a completeness check of our survey data and removed survey responses with <25% completion rate or response times of <30 seconds. No cookies were used in tracking responses. For our social media–based survey, we performed a quality analysis of the survey data and removed responses deemed suspicious for “bot” activity. We screened for suspicious responses by flagging responses with exact matching free-text responses with timestamps within a 4-hour period, and these responses were removed. We also screened for duplicate email addresses, and responses sharing the same email addresses were removed.

Secondary analyses were completed to determine if perceived knowledge of ML/AI and comfort with using ML/AI tools differed by level of provider experience or between nonproviders and providers. We also studied whether prior understanding of ML had an association with potential barriers to implementation of ML algorithms into clinical practice.

Data were analyzed using Excel (v.18.2110.13110.0; Microsoft Corporation) and SPSS Statistics (version 28; IBM Corp). Descriptive statistics were summarized as previously indicated. Independent *t* tests were used for comparison of means of each group of interest, with *t* statistic value, *df*, and the *P* value reported for each outcome. Cohen *d*_s_ (for unequal group sizes) values were calculated to estimate effect size between groups where significant differences existed. To minimize extreme responding bias and allow for binary analysis, certain Likert scale results were converted into binary format (using a score of 4-5 as a “positive” response and 1-3 as a “negative” response), which was based on similar methodology used in a previous survey-based study design [23]. Chi-square tests were used for comparing binary responses. Odds ratios with 95% confidence intervals are presented when applicable. A 2-sided α<.05 was considered significant.

### Qualitative Analysis

The free-text responses were reviewed by a member of the study team. We removed responses that were deemed to be uninterpretable (due to content unrelated to the topic or nonsensical language). The percentage of respondents who provided valid responses for our qualitative questions were determined. We performed content analysis for each free-text response for both surveys regarding concerns of use of ML/AI in practice and determined shared themes across all surveys. Each response could be categorized into one or more themes. Only themes with at least 5% of responses fitting within that category were reported.

## Results

### Single-Center Critical Care Physician Survey

**Figure 1:**
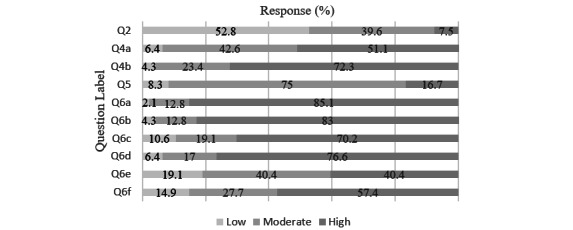
Single-center survey Likert scale results. Responses were categorized into 3 separate categories (a response of 1 or 2 was considered “low,” 3 “moderate,” and 4 or 5 “high”) and reported as percentage of valid responses out of 100%. Question content can be found in Table 2 and Multimedia Appendix 1.

Out of 79 physicians, 47 completed this internal institutional survey. The results of the survey are displayed in Tables 1 and 2 and Figure 1. All means are presented with SD. A total of 31 (59%) respondents were attendings, 19 (36%) were fellows, and 2 (4%) were residents. Perceived knowledge of ML was low (mean 2.40, SD 0.96), with 7.5% of respondents rating their knowledge as a 4 or 5. A total of 8 (15%) respondents had knowingly used an ML-based tool in their clinical practice. Confidence in predicting the need for mechanical ventilation due to COVID-19 pneumonia (mean 3.57, SD 0.79) was lower than for respiratory failure due to all other causes (mean 3.89, SD 0.78). Overall, willingness to use an ML-based algorithm was 3.32 (SD 0.95), with 75% of respondents answering 3 out of 5. Factors most likely to increase likelihood of utilization were “high quality evidence that it outperformed trained clinicians” (mean 4.28, SD 0.77), “transparency of the data utilized” (mean 4.13, SD 0.80), and “limited workflow interruption” (mean 4.09, SD 0.97), with more than 75% of respondents answering 4 or 5 for these 3 factors. For the free-response question 7 (regarding anticipated challenges with implementing an ML algorithm in practice), there were 18 (38%) out of 47 valid responses, with 2 responses removed. Shared themes and responses per theme included: accuracy/reliability (n=7, 39%), workflow interruptions/alert fatigue (n=6, 33%), patient safety (n=1, 6%), and data bias (n=2, 11%). Representative examples are shown in Multimedia Appendix 5. For question 8 regarding suggestions on ways to improve implementation, 16 (34%) out of 47 participants provided valid responses, with no responses removed. Shared themes and responses per theme included: prospective data/proof of efficacy (n=10, 63%), electronic medical record integration (n=3, 19%), and data transparency (n=3, 19%).

**Table 1:** Characteristics of respondents to a single-center survey.

Characteristics		Respondents, n (%)
**Response characteristics**		
	Response rate	53/99 (53)
	Completion rate	47/53 (89)
**Level of experience (n=53)**		
	Attending	31 (58)
	Fellow	19 (36)
	Resident	2 (3)
	Other	1 (2)
**Prior use of machine learning (n=53)**		
	Yes	8 (15)
	No	35 (66)
	Unsure	10 (18)

**Table 2:** Mean scores of Likert scale questions for a single-center survey.

Survey question		Score (1-5), mean (SD)
Q2. Level of knowledge of ML^a^		2.40 (0.968)
Q4a. Confidence in the ability to predict the need for mechanical ventilation in COVID-19		3.57 (0.801)
Q4b. Confidence in the ability to predict the need for mechanical ventilation for all other causes		3.89 (0.787)
Q5. Willingness to use ML-based tools to predict respiratory failure		3.32 (0.958)
**Factors impacting the likelihood of using the tool**		
	Q6a. High-quality evidence available	4.28 (0.772)
	Q6b. Limited workflow interruption	4.09 (0.974)
	Q6c. Transparency of the data	4.13 (0.797)
	Q6d. Real-time probability data of likelihood of need for mechanical ventilation	3.91 (0.974)
	Q6e. Support from other intensive care unit clinicians and hospital leadership	3.57 (1.12)
	Q6f. Standardized education on ML	3.34 (1.07)

^a^ML: machine learning.

### Social Media Provider and Nonprovider Survey

**Figure 2:**
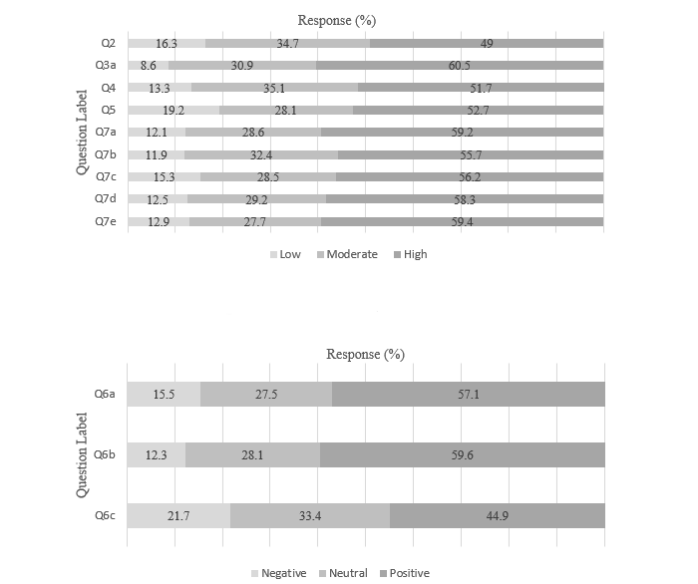
Provider survey Likert scale results. Responses were separated into 3 categories; “low,” “moderate,” or “high,” depicted in the top graph, and “negative,” “neutral,” or “positive,” depicted in the bottom graph. Results are reported as percentage of valid responses out of 100%. Question content can be found in Table 3 and Multimedia Appendix 2.

**Figure 3:**
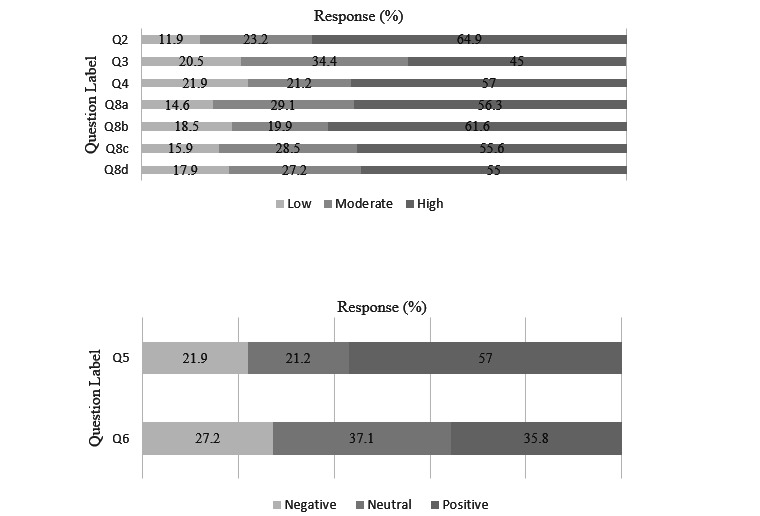
Nonprovider survey Likert scale results. Responses were separated into 3 categories; “low,” “moderate,” or “high,” depicted in the top graph, and “negative,” “neutral,” or “positive,” depicted in the bottom graph. Results are reported as percentage of valid responses out of 100%. Question content can be found in Table 4 and Multimedia Appendix 2.

We received 1196 responses, with 914 provider and 282 nonprovider responses. We excluded a total of 426 (35.6%) responses, 309 (33.8%) provider responses and 117 (41.5%) nonprovider responses. The reasons for exclusion included duplicate open-ended responses (n=324, 76.1%), duplicate email addresses (n=30, 7%), and <25% completion rate or <30-second time to completion (n=72, 16.9%). Our final analysis included a total of 770 total responses made up of 605 (78.6%) providers and 165 (21.4%) nonprovider responses. Descriptive results are displayed in Tables 3 and 4, Figures 2 and 3, and Multimedia Appendix 6. Physicians made up most of the respondents of the provider survey (n=372, 61.5%), with more attendings than medical trainees. A total of 21% (n=127) of respondents reported working in a critical care setting. Mean baseline understanding of ML/AI was 3.43 (SD 0.97), with 49% of respondents reporting a “high” level of knowledge. A total of 74% of respondents reported having used ML. Overall comfort with using an ML tool in patient care was 3.53 (SD 0.967), with 51.7% of respondents reporting a “high” level of comfort. Providers felt that efficiency and patient care were likely to benefit from these tools (means 3.68, SD 0.975 and 3.51, SD 0.938, respectively). Concern for potential negative impact on future jobs in medicine was 3.41 (SD 1.13), with 52% of respondents reporting “high” concern.

For the nonprovider survey, most nonproviders (n=90, 59%) had 1-5 encounters with the medical system in the last year. Overall confidence in physicians was 3.66 (SD 0.959), with 64.9% (n=107) reporting “high” confidence. Understanding of ML in health care was 3.03 (SD 1.23), with 45% (n=74) reporting understanding as “high” and 20.5% (n=34) as “low.” Comfort with use of ML tools in health care was 3.27 (SD 1.01), with 57% (n=94) reporting “high” level of comfort. Nonproviders overall felt positively about how ML/AI would impact medical care (mean 3.40, SD 1.01), with 57% (n=94) having a “positive” response and 21.9% (n=36) having a “negative” response. The impact on relationship with their providers was reported “positive” in 35.8% (n=59) and “negative” in 27.2% (n=45) of respondents (mean 3.09, SD 0.931). A total of 74% (n=122) of respondents would want to know if an ML algorithm was being used in their care.

For the free-text question, providers and nonproviders were asked to share any concerns they had regarding ML or AI in health care. For the providers, 312 (52%) participants of 605 provided valid responses to the free-text question, with 28 responses removed. Of the 312 total responses, 56 (16.5%) reported no concerns and 256 (75.3%) responses included a concern. Shared themes and responses per theme included the following: accuracy/reliability (n=58, 22.7%), data bias (n=35, 13.7%), patient safety/outcomes (n=34, 13.3%), doctor-patient relationship (n=28, 10.9%), privacy/security (n=22, 8.6%), workflow (n=19, 7.4%), and costs (n=14, 5.5%). Representative examples are shown in Multimedia Appendix 5. For nonproviders, 109 (66%) participants provided valid free response, with 7 responses excluded. Of those 109 valid responses, 6 (5.5%) reported no concerns and 103 (94.5%) provided concerns. Shared themes and responses per theme included the following: accuracy/reliability (n=22, 21.4%), data bias (n=22, 21.4%), privacy/security (n=16, 15.5%), patient safety/outcomes (n=11, 10.7%), lack of knowledge of ML/AI (n=11, 10.7%), and doctor-patient relationship (n=10, 10.3%). Representative examples are shown in Multimedia Appendix 5.

**Table 3:** Likert scale responses of social media survey health care provider subgroup.

Survey question		Score (1-5), mean (SD)
Q2. How would you rate your current understanding of ML^a^/AI^b^ as they apply to health care?		3.43 (0.970)
Q3a. How useful was this tool?		3.73 (0.917)
Q4. How comfortable would you feel using an ML or AI-based tool to make a clinical decision regarding your patients?		3.53 (0.967)
Q5. How concerned are you that ML/AI will make some health care jobs/specialties obsolete?		3.41 (1.13)
**Please choose the option which best describes your opinion on how the implementation of ML/AI-based tools into routine clinical practice would impact each of the following**		
	Q6a. Patient care	3.51 (0.938)
	Q6b. Efficiency in your daily practice	3.68 (0.975)
	Q6c. Patient-provider relationship	3.34 (1.054)
**Please rate the extent to which each of the following factors would increase your likelihood of using an ML or AI-based tool in your clinical practice**		
	Q7a. High-quality evidence of tool’s efficacy	3.66 (0.995)
	Q7b. Transparency of data	3.67 (1.02)
	Q7c. Workflow interruptions	3.56 (1.06)
	Q7d. Standardized education on ML/AI tools	3.63 (1.001)
	Q7e. Support from administration	3.65 (1.025)

^a^ML: machine learning.

^b^AI: artificial intelligence.

**Table 4:** Likert scale responses of social media survey nonprovider subgroup.

Survey question		Score (1-5), mean (SD)
Q2. How much confidence do you have in medical professionals’ ability to make the correct decision for your medical care?		3.66 (0.959)
Q3. How would you rate your current understanding of ML^a^ and AI^b^ as they apply to health care?		3.03 (1.233)
Q4. How comfortable would you be with having a computer algorithm using ML/AI assisting in making decisions about your medical care?		3.27 (1.013)
Q5. How do you think the implementation of more ML/AI-based algorithms into the medical system will impact your medical care?		3.40 (1.014)
Q6. How do you think the implementation of more ML/AI-based algorithms into the medical system will impact your relationship with your medical team		3.09 (0.931)
**Please rate the extent to which each of the following factors would increase your comfort level with an ML or AI-based tool being used in your medical care**		
	Q8a. High-quality evidence that it is as good or better than trained clinicians	3.65 (1.127)
	Q8b. High-quality evidence that it can improve patient outcomes	3.77 (1.214)
	Q8c. Knowing how the tool was developed	3.62 (1.082)
	Q8d. Knowing that the tool would improve efficiency	3.56 (1.141)

^a^ML: machine learning.

^b^AI: artificial intelligence.

### Secondary Analyses

In the single-center survey, there was no significant difference between critical care trainees and attendings in terms of overall perceived knowledge of ML (mean 2.19, SD 0.991 vs mean 2.50, SD 0.862; t_50_=1.19; *P*=.24) or willingness to use an ML prediction tool (mean 3.38, SD 1.05 vs 3.30, SD 0.907; t_44_=0.248; *P*=.80). For the social media survey, there was no significant difference between trainees and attending physicians in perceived knowledge (mean 3.53, SD 0.840 vs mean 3.42, SD 1.03; t_495_=1.20; *P*=.23) or comfort with ML tools (mean 3.60, SD 0.850 vs mean 3.51, SD 1.04; t_492_=0.943; *P*=.35). There was a significant difference between physician and nonprovider knowledge of ML in health care (mean 3.43, SD 0.941 vs mean 3.04, SD 1.53; t_752_=4.15; *P*<.001) and with comfort in using these tools (mean 3.53, SD 0.935 vs mean 3.28, SD 1.02; t_746_=2.90; *P*=.004). Cohen *d*_s_ values were 0.33 and 0.28, respectively, suggesting a low effect size. Comparison of critical care physicians between the 2 surveys regarding their perceived knowledge of ML revealed a significantly lower perceived knowledge among the single-center survey respondents (mean 2.40, SD 0.936 vs mean 3.27 SD 1.01; t_141_=5.08; *P*<.001). Cohen *d*_s_ value was 0.91, suggesting a large effect size. In a binary analysis of providers’ baseline knowledge (high vs low), there was not a significant association between baseline knowledge and willingness to use ML in patient care (OR 2.270, 95% CI 0.694-7.424; *P*=.17). In a binary analysis of nonproviders’ perceived knowledge of ML (high vs low), there was a significant association between higher knowledge of ML and more comfort with ML being used in patient care (OR 6.25, 95% CI 3.05-12.84; *P*<.001). The results are displayed in Table 5.

**Table 5:** Secondary analysis.

Secondary analysis subgroups			Score (1-5), mean (SD)	*t* score		*P* value^a^
**Single-center survey**						
	**Baseline knowledge of ML^b^**			–1.19		.24
		Attending	2.52 (0.991)			
		Trainee	2.19 (0.862)			
	**Willingness to use ML tool**			0.248		.81
		Attending	3.30 (0.907)			
		Trainee	3.38 (1.05)			
**Social media survey**						
	**Baseline knowledge of ML**			–1.20		.23
		Attending	3.42 (1.03)			
		Trainee	3.53 (0.840)			
	**Comfort with using ML**			0.943		.35
		Attending	3.51 (1.04)			
		Trainee	3.60 (0.850)			
	**Baseline knowledge of ML**			4.15		<.001
		Provider	3.43 (0.941)			
		Nonprovider	3.04 (1.53)			
	**Comfort with using ML tool**			2.90		.004
		Provider	3.53 (0.935)			
		Nonprovider	3.28 (1.02)			
**Cross-survey analysis**						
	**Baseline knowledge of ML**			5.08		<.001
		Critical care providers single center	2.4 (0.936)			
		Critical care providers social media	3.27 (1.01)			
**Chi-square analysis social media survey^c^**						
	Association of nonprovider comfort with using ML and baseline knowledge of ML		6.25 (3.05-12.84)	N/A^d^		<.001
	Association of provider comfort with ML and baseline knowledge of ML		2.270 (0.694-7.424)	N/A		.167

^a^Independent student *t* tests were used for comparison of means; chi-square tests were used for comparing binary responses. *P*<.05 was considered significant.

^b^ML: machine learning.

^c^Odds ratios and 95% CI are provided for this category.

^d^N/A: not applicable.

## Discussion

### Principal Findings

To our knowledge, this is the first study to explore nonprovider and provider perspectives of novel ML-based tools in critical care as well as potential implementation determinants of these tools. We found that both providers and nonproviders have favorable attitudes toward the use of ML in health care, although there remained a small but significant difference between these 2 groups with providers having more comfort overall. Nonproviders with more perceived knowledge of the concept of ML/AI were more likely to feel favorable toward its use in patient care. This finding suggests that efforts to implement ML tools may require increased focus on nonprovider education and buy-in as skepticism may be more pronounced in this group [14,24]. Second, we observed no major difference in the level of knowledge or comfort among providers regardless of their level of experience in either survey, which contradicts our preconceived notions of older providers being less comfortable with technological advancements in medicine. Third, we identified nonprovider and provider concerns about potential systemic bias in data used in ML tools, patient safety, negative effects on the doctor-patient relationship, and data privacy/security. Among providers, we also identified workflow interruptions as a major concern, and among nonproviders, limited knowledge of ML/AI was a major concern. These are critical factors that will need to be addressed to ensure user confidence in the data and algorithms. We also saw a large difference in comfort with ML among our own institution’s critical care physicians compared to the more generalized critical care physicians, suggesting that institutional differences are likely to exist and that implementation methods may need to be tailored for each institution.

Our single-center survey provided important information regarding physician acceptance of a novel algorithm for predicting the onset of mechanical ventilation in patients at risk of respiratory failure. One of the goals of this survey was to serve as a needs assessment for this tool, and based on our results, it appears that providers at our institution feel that this tool would be beneficial in the clinical context of early prediction of the need for intubation among patients with respiratory failure due to COVID-19 and all other causes [6]. These results support our team’s efforts in moving forward with the next steps of implementation, which will involve optimizing the interface for provider ease of use, preliminary prospective studies of its efficacy, and improving the sensitivity and specificity. Future steps include co-creating implementation strategies with a multidisciplinary team of clinicians, patients, implementation scientists, and medical informaticists to address identified determinants to improve the uptake and implementation of this algorithm. This process will also include additional surveys and structured interviews to assess ongoing effectiveness and to iteratively refine the algorithm to optimize its utility and improve clinical care.

### Strengths and Limitations

One of the study’s main strengths was the use of multiple platforms including social media for dissemination of our surveys, improving the generalizability of our results and allowing us to reach a large sample size. In addition, our surveys were unique in that we were able to gather both nonprovider and provider perspectives simultaneously. We also screened for suspicious responses in the social media surveys and removed these to increase the reliability of our survey results.

Despite our study’s strengths, we acknowledge the following limitations. First, due to privacy issues, we did not collect demographic or other personal data regarding the respondents. Thus, we are unable to draw conclusions regarding whether certain members of nonprovider and provider communities may be more amenable to ML methods (eg, based on gender or race). Second, our conclusions are limited to the population studied as our surveys were in English and only reached those with electronic access. Third, as with any survey, there are risks of both selection bias as well as participation bias. For selection bias in the first survey, we emailed ICU providers but did not gather any systematic data from ICU nurses or pharmacists or others who may be impacted by these tools. Regarding participation bias, it is likely that the individuals responding to social media survey would be those with an interest in this topic and thus may be more comfortable with these methods than others. Fourth, the truly open nature of the social media survey led to unanticipated issues with bot responses, and while steps were taken to remove suspicious responses, to our knowledge, there is no validated means of screening for bot activity. Fifth, there was no specific implementation conceptual framework used in the development of questions addressing implementation barriers and facilitators. Despite these limitations, we view our findings as an important step toward the successful implementation of ML/AI methods to improve patient care.

### Conclusions

Both providers and nonproviders have overall positive perspectives on the use of ML-based tools in health care, although nonproviders remain more skeptical. In addition, it appears that a tool to help predict onset of the need for intubation would be both useful and acceptable among critical care providers. Our study revealed shared concerns regarding accuracy and reliability, data bias, privacy/security, patient safety, the doctor-patient relationship, and workflow interruptions. These data provide a baseline assessment of health care provider and nonprovider perceptions of ML/AI-based tools that will be crucial in optimizing their clinical utility.

## Appendix

Multimedia Appendix 1 Single-center critical care physician survey.

Multimedia Appendix 2 Social media survey content.

Multimedia Appendix 3 Social media recruitment post examples.

Multimedia Appendix 4 Checklist for Reporting Results of Internet E-Surveys (CHERRIES).

Multimedia Appendix 5 Qualitative evaluation of provider and patient concerns regarding ML/AI in healthcare.

Multimedia Appendix 6 Demographics and categorical responses to social media survey.

## Abbreviations

AI: artificial intelligence
CHERRIES: Checklist for Reporting Results of Internet E-Surveys
ICU: intensive care unit
ML: machine learning
UCSD: University of California, San Diego

## Data Availability

Data are stored securely in Qualtrics with access restricted to University of California, San Diego users. Email addresses were removed prior to exporting data to be processed in Excel and SPSS. Data can be requested by emailing the corresponding author at emlodzinski@health.ucsd.edu.
