# Supplementary material for: Assessing Barriers to Implementation of Machine Learning and Artificial Intelligence–Based Tools in Critical Care: Web-Based Survey Study
Source: JMIR Perioper Med. 2023 Jan 27;6:e41056. doi: 10.2196/41056 (PMC10013679; doi:10.2196/41056)
Supplement: Multimedia Appendix 1 [file periop_v6i1e41056_app1.docx]

Multimedia Appendix 1. Single-Center Critical Care Physician Survey

Mechanical Ventilation Prediction Tool Questionnaire

Thank you for taking the time to participate in our survey! 

We are conducting a survey regarding a recently developed deep learning algorithm which can predict the need for mechanical ventilation in hospitalized patients with respiratory failure. We are planning to incorporate this tool into the electronic medical record (EMR) at UCSD and want to have the input of ICU clinicians to help make this tool as useful as possible. Participation in this survey is voluntary. Completion of the survey indicates your consent to participate. You may refuse to participate or withdraw at any time by simply exiting the survey. We have set up the study so that results are anonymous and your answers will not be traced to you. Research records will be kept confidential to the extent allowed by law. If you have any questions or concerns about this study, you may reach out to the principal investigator Dr. Atul Malhotra at amalhotra@health.ucsd.edu.

Q1 Please indicate your current level of training.

- Resident
- Fellow
- Attending
- Other ________________________________________________

Q2 How would you rate your current level of knowledge of **machine learning** as it applies to healthcare?

|  | 1= no knowledge | 2 | 3 | 4 | 5= very knowledgeable |
| --- | --- | --- | --- | --- | --- |
| (3) |  |  |  |  |  |

Q3 Have you ever used a **machine learning-based tool** in your clinical decision-making?

- Yes
- No
- Unsure

Display This Question:

If Have you ever used a machine learning-based tool in your clinical decision-making? = Yes

Q3a On a scale of 1-5, how useful did you the find the **machine learning-based tool?**

|  | 1= not useful at all | 2 | 3 | 4 | 5= very useful |
| --- | --- | --- | --- | --- | --- |
| (1) |  |  |  |  |  |

Q4 How confident do you feel in your ability to predict need for mechanical ventilation in the following situations?

|  | 1= not confident at all | 2 | 3 | 4 | 5= very confident |
| --- | --- | --- | --- | --- | --- |
| Patients with respiratory failure due to COVID-19 |  |  |  |  |  |
| Patients with respiratory failure due to all other causes |  |  |  |  |  |

Q5 If there were a tool available in the electronic medical record system that utilized real-time patient data to alert you to the risk of a patient requiring mechanical ventilation, how likely would you be to use such a tool in your clinical decision making?

|  | 1= would not use | 2 | 3 | 4 | 5= very likely to use |
| --- | --- | --- | --- | --- | --- |
| (1) |  |  |  |  |  |

Q6 Please rate the extent to which each of the following factors would increase your likelihood of using a machine learning-based tool to predict need for mechanical ventilation.

|  | 1= would not increase my likelihood of using the tool | 2 | 3 | 4 | 5= greatly increase my likelihood of using the tool |
| --- | --- | --- | --- | --- | --- |
| High quality evidence that it outperformed trained clinicians in ability to predict need for mechanical ventilation |  |  |  |  |  |
| Transparency of the data it utilizes in order to predict need for mechanical ventilation |  |  |  |  |  |
| Real time probability data of likelihood of need for mechanical ventilation (ie. 80% likely patient needs intubation in x hours) |  |  |  |  |  |
| Limited work-flow interruption |  |  |  |  |  |
| Standardized education on machine learning and artificial intelligence |  |  |  |  |  |
| Support for this tool from other ICU clinicians and hospital leadership |  |  |  |  |  |

Q7 Please describe any potential challenges you anticipate with the implementation of a machine learning-based predictive tool into your clinical practice.

________________________________________________________________

Q8 Please describe any other factors that you think might improve the implementation of a machine learning-based predictive tool into your clinical practice.

________________________________________________________________
