# Supplementary material for: Assessing Barriers to Implementation of Machine Learning and Artificial Intelligence–Based Tools in Critical Care: Web-Based Survey Study
Source: JMIR Perioper Med. 2023 Jan 27;6:e41056. doi: 10.2196/41056 (PMC10013679; doi:10.2196/41056)
Supplement: Multimedia Appendix 2 [file periop_v6i1e41056_app2.docx]

Multimedia Appendix 2. Social Media Survey Content

We are conducting this survey in order to gather information on health care providers’ and patients’ comfort with the use of machine learning (ML) and artificial intelligence (AI) in medical care. Machine learning is a form of artificial intelligence that uses computer algorithms to analyze and learn from large collections of data in order to make predictions/decisions without the need for direct input from humans. 

Participation in this survey is voluntary. Completion of the survey indicates your consent to participate. You may refuse to participate or withdraw at any time by simply exiting the survey. We have set up the study so that results are anonymous and your answers will not be traced to you. You have the option to provide your email address at the end of the survey for us to reach out for future surveys and for a chance to win a prize. This is not required, and if provided, your email address will not be shared with any third parties or used for any additional purposes other than those described. Research records will be kept confidential to the extent allowed by law.

Are you a health care provider? (MD, DO, MBBS, PA, NP, RN, or student/in-training)

- Yes
- No

*(If yes, will bring respondent to Survey 1. If no, will bring respondent to survey 2)*

*Health Care Provider Subgroup Survey*

Please choose the option which best describes your role in healthcare

- Medical Student
- Medical Resident or Fellow
- Attending Physician
- Nurse
- Advanced practice provider
- Other (Please describe)

________________________________________________

How would you rate your current understanding of machine learning (ML) and artificial intelligence (AI) as they apply to healthcare?

- 1= no knowledge
- 2
- 3
- 4
- 5= very knowledgeable

Have you ever used a ML or AI-based decision tool in your clinical practice?

- Yes
- No
- Unsure

If yes, how useful was this tool?

- 1= not useful at all
- 2
- 3
- 4
- 5= very useful

How comfortable would you feel using an ML or AI-based tool to make a clinical decision regarding your patients?

- 1= very uncomfortable
- 2
- 3
- 4
- 5= Very comfortable

Please choose the option which best describes your opinion on how the implementation of ML/AI-based tools into routine clinical practice would impact each of the following.

|  | 1= very negatively impact | 2= somewhat negatively impact | 3= no impact | 4= somewhat positively impact | 5= very positively impact |
| --- | --- | --- | --- | --- | --- |
| Patient care and patient outcomes |  |  |  |  |  |
| Efficiency in your daily practice |  |  |  |  |  |
| The provider-patient relationship |  |  |  |  |  |

How concerned are you that ML/AI will make some health care jobs/specialties obsolete?

- 1= Not concerned at all
- 2
- 3
- 4
- 5= Very concerned

Please rate the extent to which each of the following factors would increase your likelihood of using an ML or AI-based tool in your clinical practice

|  | 1= would not increase my likelihood of using the tool | 2 | 3 | 4 | 5= would greatly increase my likelihood of using the tool |
| --- | --- | --- | --- | --- | --- |
| High quality evidence that it outperformed trained clinicians |  |  |  |  |  |
| Transparency of the data it utilizes in order to predict the outcome of interest |  |  |  |  |  |
| Limited work-flow interruption |  |  |  |  |  |
| Standardized education on machine learning and artificial intelligence |  |  |  |  |  |
| Support for this tool from other clinicians and hospital leadership |  |  |  |  |  |

Please choose your main specialty/practice setting

- Anesthesiology
- Critical Care Medicine (including pulmonary, anesthesia, surgical, neuro, and pediatric critical care medicine)
- Dermatology
- Emergency Medicine
- Family Medicine
- Internal Medicine (including subspecialties not otherwise listed)
- Neurology
- Pathology
- Pediatrics (including subspecialties not otherwise listed)
- Psychiatry
- Radiology
- Student
- Surgery (including subspecialties not otherwise listed)
- Other (please describe)

________________________________________________

Please describe any concerns you may have about utilizing ML and AI based tools in your clinical practice.?

________________________________________________________________

Would you be interested in being contacted for follow up surveys or individual interviews regarding this topic? If so, please provide an email address where we can contact you. You will also be entered for a chance to win an amazon gift card!

________________________________________________________________

We are also performing another survey regarding patient perspectives on ML/AI in medicine. If you are interested in sharing your perspective as a potential patient, please **click here** to complete an additional survey with an additional chance to win a prize!

*Patient Subgroup Survey*

In the last year, how many times have you visited a healthcare provider (including office visits, emergency room visits, and hospitalizations)

- 0
- 1-5
- 6-10
- 11+

In general, how much confidence do you have in medical professionals' ability to make the correct decision for your medical care?

- 1= None at all
- 2= A little
- 3= A moderate amount
- 4= A lot
- 5= A great deal

How would you rate your current understanding of machine learning (ML) and artificial intelligence (AI) as they apply to healthcare?

- 1= no knowledge
- 2
- 3
- 4
- 5= very knowledgeable

How comfortable would you be with having a computer algorithm using ML/AI assisting in making decisions about your medical care?

- 1= Very uncomfortable
- 2= Somewhat uncomfortable
- 3= Neither comfortable nor uncomfortable
- 4= Somewhat comfortable
- 5= Extremely comfortable

How do you think the implementation of more ML/AI-based algorithms into the medical system will impact your medical care?

- 1= very negatively impact
- 2= somewhat negatively impact
- 3= no impact
- 4= somewhat positively impact
- 5= very positively impact

How do you think the implementation of more ML/AI-based algorithms into the medical system will impact your relationship with your medical team?

- 1= very negatively impact
- 2= somewhat negatively impact
- 3= no impact
- 4= somewhat positively impact
- 5= very positively impact

Would you want to know whether AI/ML was being used in your medical care?

- Yes
- No
- Unsure

Please rate the extent to which each of the following factors would increase your comfort level with an ML or AI-based tool being used in your medical care

|  | 1= would not increase my comfort level | 2 | 3 | 4 | 5= would greatly increase my comfort level |
| --- | --- | --- | --- | --- | --- |
| High quality evidence that it is as good or better than trained clinicians |  |  |  |  |  |
| High quality evidence that it can improve patient outcomes |  |  |  |  |  |
| Knowing how the tool was developed (ie, type of data used for development, access to validation studies for the tool) |  |  |  |  |  |
| Knowing that the tool would improve efficiency |  |  |  |  |  |

Please describe any concerns you may have about ML or AI-based tools in healthcare.

________________________________________________________________

Would you be interested in being contacted for follow up surveys or individual interviews regarding this topic? If so, please provide an email address where we can contact you. You will also be entered for a chance to win an amazon gift card!

________________________________________________________________
