# Supplementary material for: Assessing Barriers to Implementation of Machine Learning and Artificial Intelligence–Based Tools in Critical Care: Web-Based Survey Study
Source: JMIR Perioper Med. 2023 Jan 27;6:e41056. doi: 10.2196/41056 (PMC10013679; doi:10.2196/41056)
Supplement: Multimedia Appendix 3 [file periop_v6i1e41056_app3.docx]

Multimedia Appendix 3. Social Media Recruitment Post Examples

Twitter Post

Calling all healthcare providers and patients! Please help us by sharing your thoughts on #AI and #MachineLearning in healthcare. Complete this IRB approved 5 min. survey (<https://bit.ly/MLSurvey1>) and be entered to win a $100 Amazon gift card! Please RT! #UCSDHealth

Facebook Post

Our research team at [UC San Diego Health](https://www.facebook.com/UCSDHealth?__cft__%5b0%5d=AZXmRO2V16KTgl5e-RDocxLRrtwYkVpP4jQlxan_RW5MtgFF1HviJ3ave3OkSA5kGTLcHOmXa7mnN8bP4V8L50ixEu3nfU34RWIaxvo0mp_0bi7ZDknY5UanJvzrjja8ycjoXdtnBA3rsOG-6Jij0w4EiK-deuW03qgtErgnrjxrhOErE2-8m36n1cwexrPVPtHZDJXlR75GRpeczb3Slbor&__tn__=-%5dK*F) is evaluating patient and healthcare provider perspectives on machine learning and artificial intelligence in healthcare. Take our super short (<5 minute) survey: [https://bit.ly/MLAISurvey](https://l.facebook.com/l.php?u=https%3A%2F%2Fbit.ly%2FMLAISurvey%3Ffbclid%3DIwAR2N_jwcDEpqwGu1y8feXL-80nx-VQjadfYXj3XNfGljZcICviFpc2Usu1U&h=AT0pjo4lxStWy3onsD42dmzR23h03P5jNnYfL9OUnf7smVmidoWqFOsZN8eWfedgi-Eu7bcLQ9A8WKBUyJVAFMMEmQz69cD1CqhZCceU2-HD5uxYNSiHR2M3fMlNl_uZGyi7p_VieQdYmziIQA&__tn__=-UK*F&c%5b0%5d=AT2mwGWhRhpmcITdhDgczrmMy4bqq1TRMzBEXPnOrNangzPTt41YsivMICoqWAY1OKbjlHLmg35aGL68lmKQ45LeQGym88H4zMWX6Wa5FaqD0l4rZ0alKK2HgoDXRXeHxAAZd2LZxkY3PBgtcNxbtAI85Alc_ZG5G9ur4mi2J9Q2JrJrAoJ5phchMZdPHLxNBmfcn3h6TcKEPAdal1t-V6gYK9yYQ8El5a4). Plus, you will be entered to win a $100 gift card. Please share! [#patients](https://www.facebook.com/hashtag/patients?__eep__=6&__cft__%5b0%5d=AZXmRO2V16KTgl5e-RDocxLRrtwYkVpP4jQlxan_RW5MtgFF1HviJ3ave3OkSA5kGTLcHOmXa7mnN8bP4V8L50ixEu3nfU34RWIaxvo0mp_0bi7ZDknY5UanJvzrjja8ycjoXdtnBA3rsOG-6Jij0w4EiK-deuW03qgtErgnrjxrhOErE2-8m36n1cwexrPVPtHZDJXlR75GRpeczb3Slbor&__tn__=*NK*F) [#machinelearning](https://www.facebook.com/hashtag/machinelearning?__eep__=6&__cft__%5b0%5d=AZXmRO2V16KTgl5e-RDocxLRrtwYkVpP4jQlxan_RW5MtgFF1HviJ3ave3OkSA5kGTLcHOmXa7mnN8bP4V8L50ixEu3nfU34RWIaxvo0mp_0bi7ZDknY5UanJvzrjja8ycjoXdtnBA3rsOG-6Jij0w4EiK-deuW03qgtErgnrjxrhOErE2-8m36n1cwexrPVPtHZDJXlR75GRpeczb3Slbor&__tn__=*NK*F) [#Healthcare](https://www.facebook.com/hashtag/healthcare?__eep__=6&__cft__%5b0%5d=AZXmRO2V16KTgl5e-RDocxLRrtwYkVpP4jQlxan_RW5MtgFF1HviJ3ave3OkSA5kGTLcHOmXa7mnN8bP4V8L50ixEu3nfU34RWIaxvo0mp_0bi7ZDknY5UanJvzrjja8ycjoXdtnBA3rsOG-6Jij0w4EiK-deuW03qgtErgnrjxrhOErE2-8m36n1cwexrPVPtHZDJXlR75GRpeczb3Slbor&__tn__=*NK*F)
