# Supplementary material for: Assessing Barriers to Implementation of Machine Learning and Artificial Intelligence–Based Tools in Critical Care: Web-Based Survey Study
Source: JMIR Perioper Med. 2023 Jan 27;6:e41056. doi: 10.2196/41056 (PMC10013679; doi:10.2196/41056)
Supplement: Multimedia Appendix 5 [file periop_v6i1e41056_app5.docx]

Multimedia Appendix 5. Qualitative Evaluation of Provider and Non-Provider Concerns Regarding ML/AI in Healthcare

| Category of Shared Concern | Free Responses |
| --- | --- |
| Providers |  |
| Accuracy/Reliability | “Reliability, accuracy and trustworthiness.”  “Depends on how reliable such a tool is. e.g. if the tool has a 20% in accuracy rate then anyway we will be relying on our clinical judgement to determine which patients will need intubation. So this extra machine learning tool would not add any benefit in such a situation.” |
| Workflow | “Should improve EMR efficiency, will not benefit if takes longer when using EMR.”  “Complicating workflow and potentially worsening alarm/alert fatigue.” |
| Privacy/Security | “Privacy is one of the main threats in the development and utilization of data resources. Intelligent systems have a large amount of personal information. If some private information is used illegally for commercial purposes, it will cause data leakage and privacy infringement.”  “There are big loopholes in system security, which can be easily invaded by hackers and damage personal privacy and property security.” |
| Patient Safety/Outcomes | “Liability for decisions made if it is not considered standard of care.”  “Technical defects may lead to abnormal operation and potential safety risks.” |
| Data Bias | “That data used to inform AI / ML will perpetuate biases in the data.”  “Concern about bias in source dataset contributing to ongoing health disparities.”  “A lot of the training data has bias baked into it, and algorithm use is often viewed as unbiased. This has the risk of codifying existing bias but being viewed as unbiased.” |
| Patient Distrust/Doctor-Patient Relationship | “AI has some difficulties in communicating with patients and some family members of patients do not understand and distrust AI.”  “The main concern is that most patients still do not trust robots, which will affect the doctor-patient relationship.” |
| Cost | “The ever-increasing cost of delivering health care is a concern. I would want to see a clear outcome benefit as I am assuming this technology is not cheap.” |
| Non-Providers |  |
| Accuracy/Reliability | “Blind acceptance/trust in AI/ML results without checks or verification of accuracy/applicability.”  “I am worried it is not accurate.” |
| Data Bias | “Is the data being used in the algorithm equitable, representative of me?”  “We know that there are worries about such algorithms further disadvantaging already disadvantaged groups, and that many algorithms are biased. For this reason, at the very least it should be mandatory for patients to be made aware if AI technology is used in patient care.”  “Bias, both specifically racial implications and broadly with incomplete information having doctors not being asked to engage and think critically about treating patients and instead reliant on incomplete AI information.” |
| Patient Safety/Outcomes | “Improper use of medical risks, and safety risks.” |
| Privacy/Security | “I think it's going to affect my privacy, because ai is going to store my medical data.”  “Criminals could take advantage of various security risks of artificial intelligence.” |
| Lack of Knowledge | “Need to learn more about ML.”  “I do not know much about how it works or whether it works.” |
| Patient-Provider Relationship | “Further deepen the contradiction between doctors and patients.”  “Forgetting about the patient and only looking at the data.” |

EMR= Electronic Medical Record; ML= Machine Learning; AI= Artificial Intelligence
