# Supplementary material for: Assessing Barriers to Implementation of Machine Learning and Artificial Intelligence–Based Tools in Critical Care: Web-Based Survey Study
Source: JMIR Perioper Med. 2023 Jan 27;6:e41056. doi: 10.2196/41056 (PMC10013679; doi:10.2196/41056)
Supplement: Multimedia Appendix 6 [file periop_v6i1e41056_app6.docx]

Multimedia Appendix 5. Demographics and Categorical Responses to Social Media Survey

| Survey Question | | Frequency (n) | | Percent |
| --- | --- | --- | --- | --- |
| Are you a healthcare provider? | |  |  | |
| Yes | 605 | | 78.6 | |
| No | 165 | | 21.4 | |
|  |  | |  | |
| *Provider Subgroup* |  | |  | |
| Level of Training |  | |  | |
| Medical Student | 126 | | 20.9 | |
| Medical Resident or Fellow | 173 | | 26.6 | |
| Attending | 199 | | 32.9 | |
| Nurse | 64 | | 10.6 | |
| Advanced Practice Provider | 31 | | 5.1 | |
| Other | 11 | | 1.8 | |
| Medical Specialty |  | |  | |
| Anesthesiology | 19 | | 3.1 | |
| Critical Care | 127 | | 21 | |
| Dermatology | 51 | | 8.4 | |
| Emergency Medicine | 87 | | 14.4 | |
| Family Medicine | 58 | | 9.6 | |
| Internal Medicine | 101 | | 16.7 | |
| Neurology | 15 | | 2.5 | |
| Pathology | 22 | | 3.6 | |
| Pediatrics | 33 | | 5.5 | |
| Psychiatry | 15 | | 2.5 | |
| Radiology | 10 | | 1.7 | |
| Student | 25 | | 4.1 | |
| Surgery | 29 | | 4.8 | |
| Other | 12 | | 2 | |
|  |  | |  | |
| Have you ever used a ML or AI-based decision tool in your clinical practice? |  | |  | |
| Yes | 447 | | 74 | |
| No | 118 | | 19.5 | |
| Unsure | 39 | | 6.5 | |
| *Patient Subgroup* |  | |  | |
| In the last year, how many times have you visited a healthcare provider? |  | |  | |
| 0 | 13 | | 8.6 | |
| 1 to 5 | 90 | | 59.2 | |
| 6 to 10 | 39 | | 25.7 | |
| 11+ | 10 | | 6.6 | |
|  |  | |  | |
| Would you want to know whether AI/ML was being used in your medical care? |  | |  | |
| Yes | 113 | | 74.3 | |
| No | 18 | | 11.8 | |
| Unsure | 24 | | 15.8 | |
|  |  | |  | |

ML= Machine Learning; AI= Artificial Intelligence
